# Morphological and histological changes in the urethra after intraurethral nonablative erbium YAG laser therapy: an experimental study in beagle dogs

**DOI:** 10.1007/s10103-022-03575-3

**Published:** 2022-05-26

**Authors:** Sheng-fei Xu, Kuerbanjiang Abulikim, Xiao-yu Wu, Yu Cheng, Qing Ling, Ke Rao, Kai Cui, Zhong Chen, Guang-hui Du, Xiao-yi Yuan

**Affiliations:** Department of Urology, Tongji Hospital, Tongji Medical College, Huazhong University of Science and Technology, No.1095 Jie Fang Avenue, Hankou, Wuhan, Hubei Province 430030 People’s Republic of China

**Keywords:** Er:YAG laser, Urethra, Stress urinary incontinence, TGF-β1, α-SMA, Laser therapy

## Abstract

The purpose of this study was to investigate the morphological and histological changes in the urethra in beagle dogs after intraurethral Er:YAG laser irradiation in nonablative mode to confirm the safety of this therapy. Six 2-year-old healthy female virgin beagle dogs (13 ± 1.51 kg) were used in this study. The animals were divided into 2 groups: the sham group, which received sham treatment (*n* = 3) involving insertion of an intraurethral cannula and laser delivery handpiece into the urethra without laser irradiation, and the experimental group (*n* = 3), which received intraurethral Er:YAG laser irradiation. The laser irradiation parameters were set according to clinical criteria (4 mm spot size, 1.5 J/cm^2^, 1.4 Hz, and 4 pulses) in nonablative mode. All animals received three sequential sessions at 4-week intervals. Urethrography and urethroscopy were performed in the 12th week and 13th week, respectively, after the first treatment. After urethroscopy, the animals were sacrificed, and urethral tissue was harvested for histological investigations. All procedures were performed under general anesthesia (40 mg/kg 3% sodium pentobarbital, i.v.). Transforming growth factor β1 (TGF-β1) and α-smooth muscle actin (α-SMA) expression levels were measured to evaluate the biochemical characteristics of the scar. Urethral stricture was not found by urethrography or urethroscopy in either group. Urethral epithelium thickness and collagen expression under the urethral mucosa were significantly increased in the experimental group compared with the sham group. However, there were no significant differences in TGF-β1 and α-SMA expression between the experimental group and sham group (*p* > 0.05). Urethral stricture is not found in beagle dogs after clinically relevant intraurethral nonablative mode Er:YAG laser irradiation. Proliferation of urethral collagen and the urethral mucosa may be one of the mechanisms by which urine leakage symptoms can be improved.

## Introduction

According to the International Continence Society (ICS) and the International Urogynecology Association (IUGA), urinary incontinence (UI) is defined as involuntary loss of urine and is a common form of urinary dysfunction experienced by adult females [1]. UI is categorized as stress urinary incontinence (SUI), urgency urinary incontinence (UUI), and mixed-type urinary incontinence (MUI). Nearly 50% of adult women experience urinary incontinence,[2, 3] and approximately 37.5% of young women (30–50 years) complain of SUI [4], which can seriously affect the health and quality of life of women [3, 5]. Treatments for female SUI are mainly classified as conservative or surgical [3]. At present, for most patients with SUI, the initial treatment option is conservative treatment, such as pelvic floor muscle training (PFMT), electrical stimulation, biofeedback, or medications (e.g., duloxetine) [3, 6]. However, the effectiveness of these treatments relies on adherence to treatment [7–9]. For patients with severe leakage symptoms or for whom conservative treatment fails, surgical treatment may be offered. Surgical treatment options include midurethral slings, Burch colposuspension, urethral bulking, and artificial urinary sphincter [3, 10]. Although surgical methods, especially midurethral sling, can be efficient, they can also lead to a range of adverse effects and complications, such as bleeding, infection, bladder perforation, mesh exposure, and groin pain [11, 12]. Due to the increasing number of reports of these adverse events, the number of sling procedures has obviously decreased in recent years [13–15]. Thus, patients are seeking alternative less invasive or noninvasive procedures.

In the past decade, many clinical studies have reported that nonablative mode erbium:yttrium aluminum garnet (Er:YAG) laser therapy can effectively treat female SUI [16–20]. The mechanism of this treatment is based on collagen remodeling and reconstruction, which strengthen the vaginal wall and improve SUI symptoms by enhancing urethral support [19–21]. Recently, innovative intraurethral Er:YAG laser therapy in nonablative mode alone was reported to relieve female SUI symptoms, and the preliminary results showed that it is a promising treatment for this disease [16]. However, the safety of this treatment [16, 22, 23] and the mechanism of its effects have not yet been clarified.

This study aimed to investigate the morphological and histological changes in the urethra in beagle dogs after intraurethral Er:YAG laser irradiation in nonablative mode to confirm the safety of this therapy and to explore the therapeutic mechanism.

## Materials and methods

### Animals

Six 2-year-old, healthy, female, virgin, beagle dogs (13 ± 1.51 kg) were purchased from the Laboratory Animal Center of Tongji Hospital, Tongji Medical College, Huazhong University of Science and Technology (Wuhan, China) in this study. To be included in the study, dogs had to be clinically healthy with no signs of reproductive system diseases. All dogs were housed in separate cages with a controlled temperature of 22–24 °C and maintained on a constant 12-h dark/12-h light cycle. The animals were provided free access to food and water. All procedures were performed under general anesthesia (40 mg/kg 3% sodium pentobarbital, i.v.), and every effort was made to minimize animal suffering. At the end of the experiment, all dogs were sacrificed by injection of an overdose of sodium pentobarbital, and tissue sections were obtained. The animal experiments were conducted in accordance with local protocols for the care and use of laboratory animals. The animal care and use protocols were approved by the Ethics Committee on Animal Experiments of Tongji Hospital, Tongji Medical College, Huazhong University of Science and Technology (TJH-201903001).

### Experimental design

**Fig. 1:**
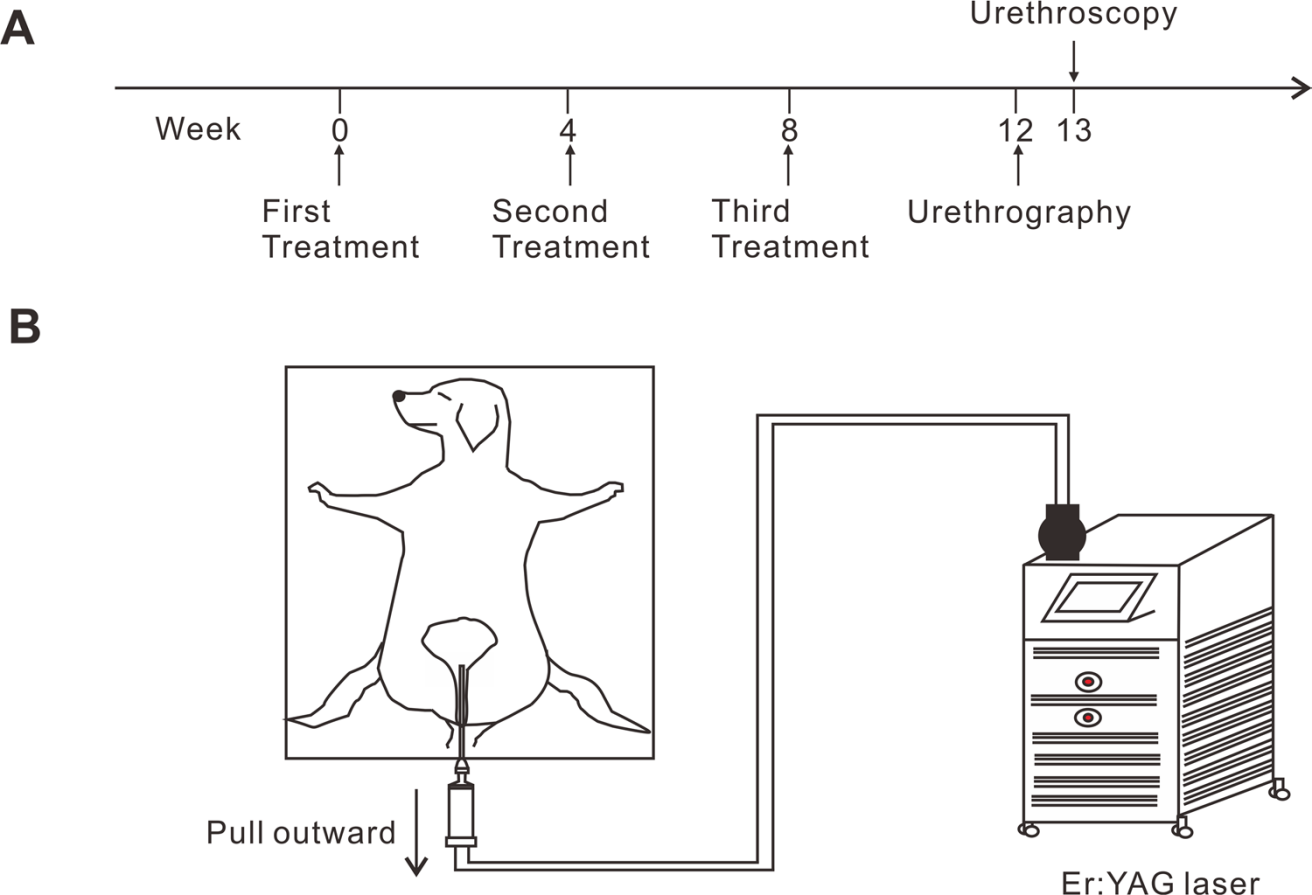
Animal experiment schedule and schematic. **A** Animal experiment schedule. **B** Schematic of the animal experiment

The animals were randomly allocated by drawing lots into two groups: the control group (*n* = 3), which received sham treatment involving insertion of an intraurethral cannula and laser delivery handpiece into the urethra without laser irradiation, and the experimental group (*n* = 3), which received intraurethral nonablative mode Er:YAG laser irradiation. All animals underwent three sequential sessions at 4-week intervals. Urethrography and urethroscopy were performed 4 weeks and 5 weeks after the last session, respectively (Fig. 1A). No animal exclusions during the experiments were made in this study.

### Intraurethral therapy

In this experiment, an intraurethral cannula (R09-2Gu, Fotona, Slovenia) was used [16]. The length of the cannula is 15 cm, and the surface has 22 scales engraved on the cannula, each scale is 5 mm apart. The Er:YAG laser beam is introduced into the cannula, then the laser beam leaves the cannula diverges to provide circular homogeneous coverage of the urethral wall.

After anesthesia, the animals were placed in the supine position, and the vulva was disinfected with iodophor and cushioned with surgical towels. Insert a ureteroscope to measure the distance between the external urethral orifice and the bladder neck. Immediately before laser treatment, urine was evacuated from the bladder using an 8F silicone catheter. Set the laser system to the intraurethral mode.

All animals in the experimental group were irradiated with 4 pulses with a fluence of 1.5 J/cm^2^ at 1.4 Hz using a nonablative mode Er:YAG laser (FotonaSmooth™ SP, Fotona, Slovenia). Laser treatment of the urethral mucosa starts from the proximal end of urethra and continues towards its orifice. With the cannula fully inserted, the first burst of four SMOOTH laser pulses was delivered at the proximal end of the urethra. After that, the cannula is pulled outwards by 5 mm according to the scale engraved on the cannula, and the next burst of four SMOOTH pulses is delivered. The process is repeated until the distal end of urethra is reached. After the completion of the first full pass, reinsert the cannula all the way towards the proximal end of urethra and repeat the procedure, delivering four SMOOTH pulses every 5 mm. Repeat the procedure until four full treatment passes are completed, and finally, removal of the intraurethral cannula. After therapy, an antibiotic (cefdinir, 100 mg) was orally administered to the animal once a day for 3 days. The animals in the control group underwent the same procedure as those in the experimental group except that the laser system was turned off.

### Urethrography

Four weeks after the last intraurethral treatment, animals were assessed by urethrography. The animals were placed in the supine position on a C-arm fluoroscope (DR 7100, Kodak, USA) under general anesthesia. An 8F silicone catheter was inserted into the urethra, and urethrography was performed under fluoroscopy after iohexol (Beilu, Beijing, China) was injected into the catheter.

### Urethroscopy

One week after urethrography, the animals were transferred to the operating room and fixed in the supine position under general anesthesia. After vulva disinfection with iodophor, a 9.5F rigid ureteroscope (Karl-Storz, Tuttlingen, Germany) was advanced into the urinary bladder through the urethra, and the morphology of both the urethral mucosa and urethral cavity morphology was assessed.

### Histological analysis and immunofluorescence (IF)

After urethroscopy, the six dogs were sacrificed by injection of an overdose of sodium pentobarbital. Urethral specimens embedded in optimal cutting temperature compound were snap-frozen on dry ice and stored at − 80 °C. The proximal part of each urethral specimen was transversely cut into 8-μm sections and stained with hematoxylin and eosin (H&E), Masson’s trichrome (MT) staining, and IF staining. IF staining was performed using monoclonal antibodies against α-smooth muscle actin (α-SMA) and transforming growth factor β1 (TGF-β1).

For IF staining, frozen slides were baked in a 37 °C oven for 10–20 min to remove moisture and fixed in paraformaldehyde for 30 min. Thereafter, the slides were immersed in EDTA antigen retrieval buffer (pH 8.0), incubated at subboiling temperature for 8 min, incubated for 8 min, and then incubated at subboiling temperature for another 7 min. Then, the tissues were incubated 3% bovine serum albumin for 30 min to block nonspecific binding.

After blocking, the sections were washed with 0.1 M PBS and incubated overnight at 4 °C with a rabbit anti-transforming growth factor β1 (TGF-β1) antibody (1:100; Servicebio, Hubei, China) or a mouse anti-α-smooth muscle actin (α-SMA) antibody (1:500; Servicebio, Hubei, China). Thereafter, the sections were washed with 0.1 M PBS and incubated with a goat anti-rabbit IgG conjugated to CY3 (1:300; Servicebio, Hubei, China) or anti-mouse IgG conjugated to CY3 (1:300; Servicebio, Hubei, China) at room temperature for 50 min. The nuclei were stained with DAPI (Servicebio, Hubei, China). Thereafter, the sections were washed three times with 0.1 M PBS for 5 min. Then, AutoFluo Quencher (Servicebio, Hubei, China) was applied for 5 min, and the sections were washed with running tap water for 10 min. Finally, the sections were dried and sealed with antifade mounting medium (Servicebio, Hubei, China).

For image analysis, four randomly selected fields per tissue were photographed using fluorescence microscopy (Eclipse C1, Nikon, Japan). The submucosal region in which collagen was distributed was selected as the field of interest. For each selected field, the positive area was calculated with ImageJ software (version 1.52v, NIH, USA). The results are expressed as the volume fraction (percentage of positive area in relation to the total area). TGF-β1 and α-SMA levels in the submucosa of the urethra (excluding the urethral mucosa area) were measured in a similar manner as collagen levels. In addition, we randomly measured the thickness of urethral epithelial cells in the 3, 6, 9, and 12 o’clock positions in the urethra.

### Statistical analysis

The values for each parameter were averaged for each animal, and the quantitative data are expressed as the means ± standard deviation (SD). Statistical analysis of differences between groups was performed using SigmaPlot (version 14, Systat Software, Inc., USA). The data were compared using two-tailed Student’s t-test. A *p* value of less than 0.05 was considered to indicate a significant difference between groups.

## Results

### Urethrography

**Fig. 2:**
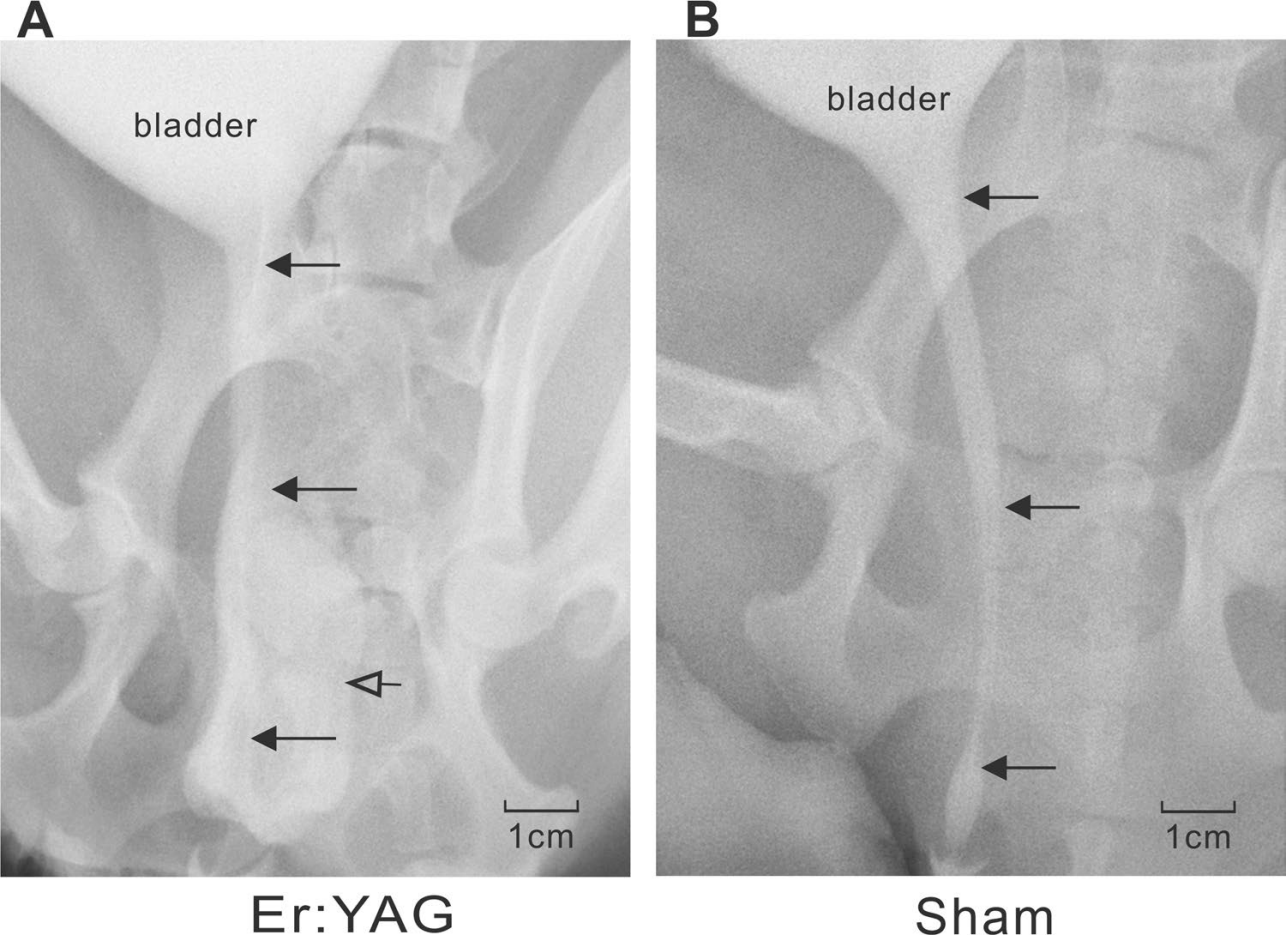
Urethrography. Urethrography revealed maintenance of a wide urethral caliber without stricture in both the experimental (**A**) and sham (**B**) groups at 4 weeks post-treatment. Solid arrow, urethra; hollow arrow, vagina

The urethrograms showed that the urethral caliber of the dogs in the experimental group was similar to that of the dogs in the sham group, which indicated that experimental dogs maintained a wide urethral caliber without any sign of stricture 4 weeks post-treatment (Fig. 2).

### Urethroscopy assessment

**Fig. 3:**
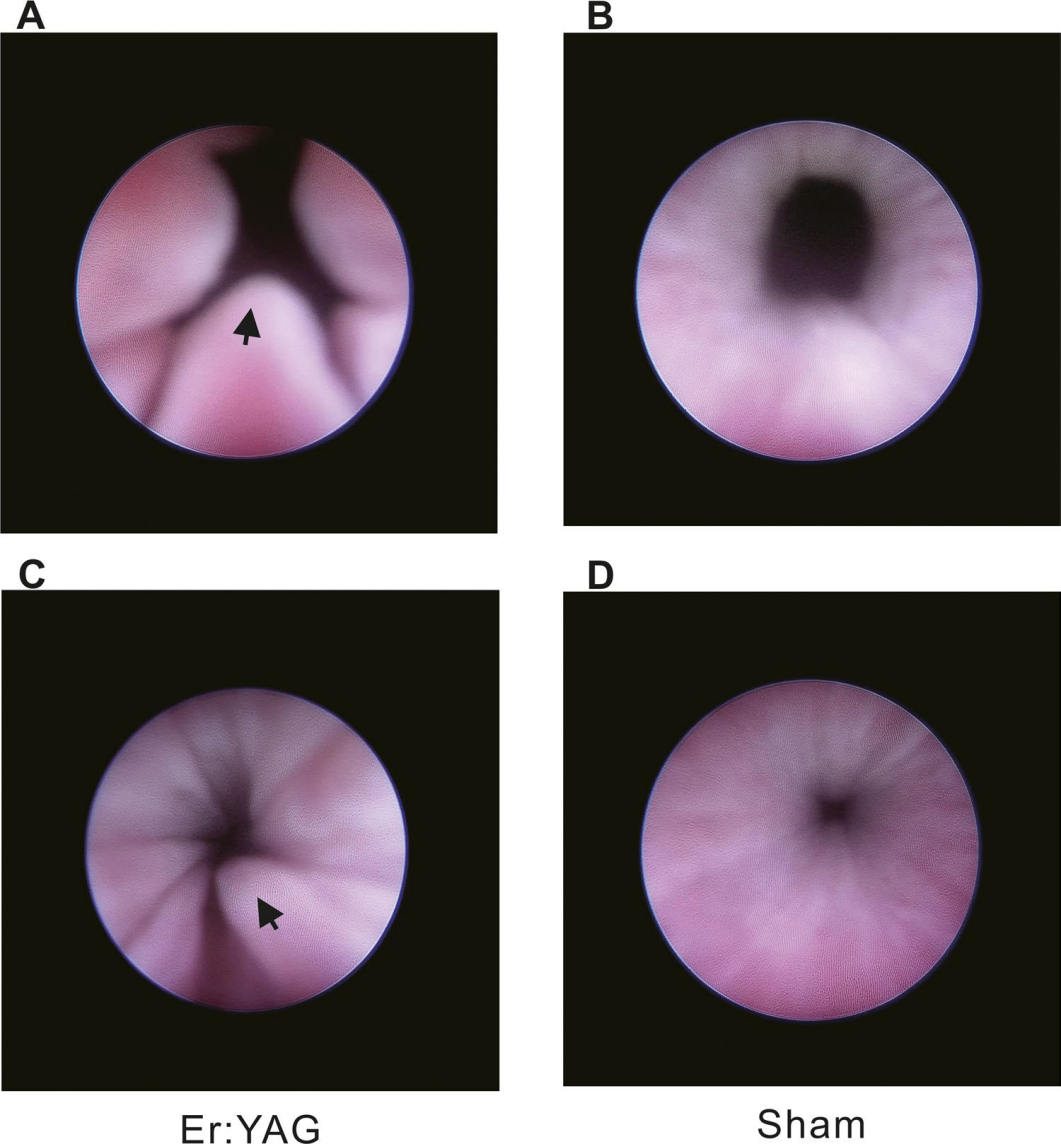
Comparison of the condition of the urethral lumen in beagle dogs using urethroscopy after intraurethral treatment. **A** bladder neck and **C** middle urethra of the Er:YAG laser irradiation group. **B** bladder neck and **D** middle urethra of the sham group. Solid arrow, the urethral mucosa hyperplasia, manifested as increased mucosal folds

Urethral mucosa proliferation, which manifested as increases in mucosal folds, was observed in all dogs in the experimental group. Urethral stricture was not observed at 5 weeks post-treatment (Fig. 3). In contrast, in the dogs in the sham group, the urethral mucosa was smooth, and there were few urethral mucosal folds (Fig. 3).

### Histology

At low magnification, H&E-stained sections from the experimental group showed a roughly normal urethral lumen with increased urethral mucosal plica. In the sham group, H&E-stained urethral cross-sections revealed a smooth urethral mucosa and fewer urethral mucosal folds.

**Fig. 4:**
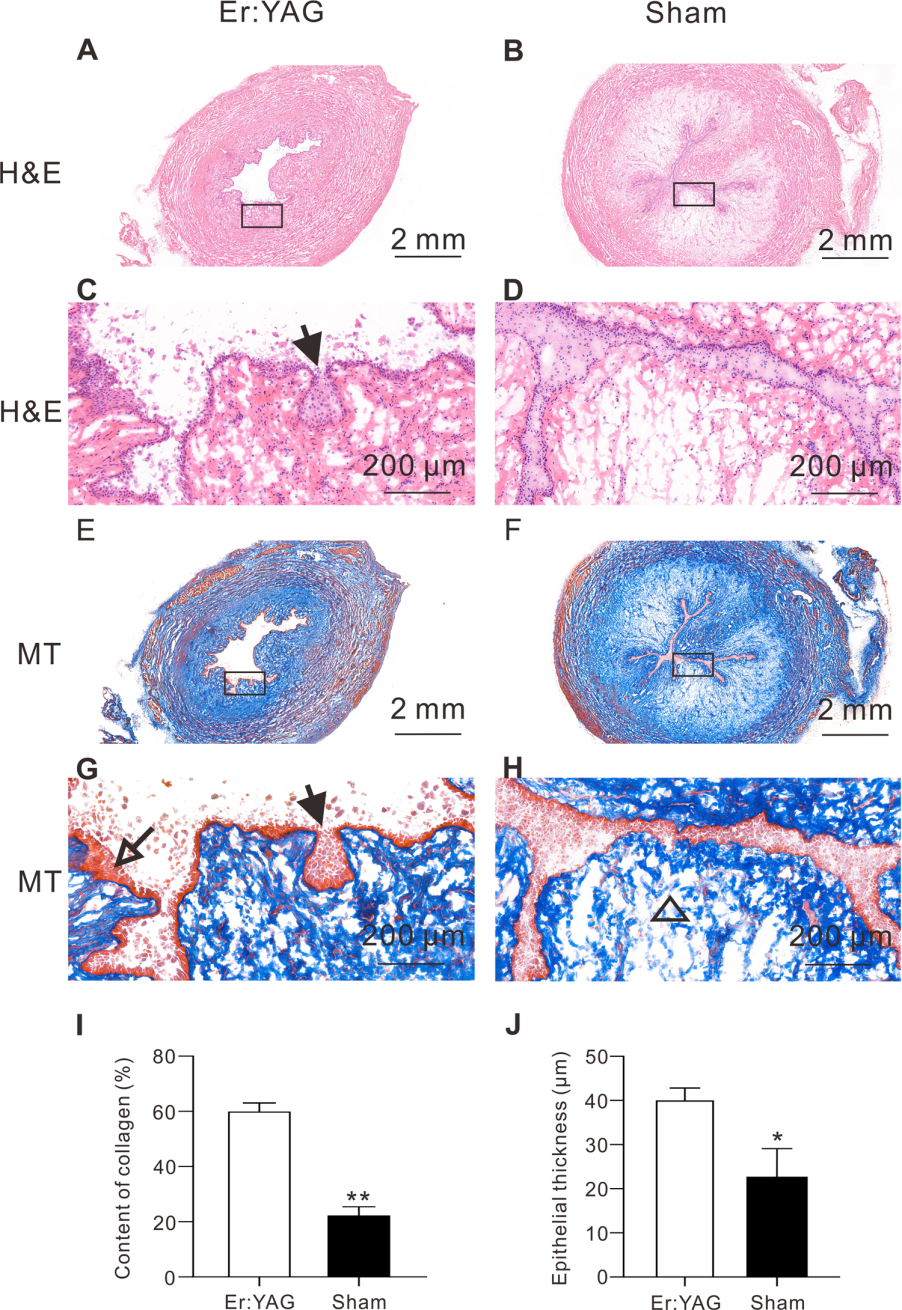
H&E and MT staining of the urethral mucosa at 5 weeks post-treatment. Solid arrow, the urethral mucosa became more wrinkled after Er:YAG laser irradiation (**C** and **G**). Hollow arrow, the urethral mucosa was thicker on average after Er:YAG laser irradiation (**G**). Hollow triangle, MT staining of the urethral mucosa resulted in lighter blue staining in sham group than in the Er:YAG laser group, indicating low collagen deposition (**H**). Graph (**I** and **J**): quantitative analysis of the content of collagen and epithelial thickness in urethral tissue sections, respectively (mean ± SD, *p < 0.05, **p < 0.001)

At high magnification, both groups exhibited a stratified columnar epithelium with a complete basement membrane. There was no tissue contraction or scar tissue hyperplasia in the urethral mucosa in either group. In the experimental group, the urethral epithelial cell layer was thicker on average after Er:YAG laser irradiation (39.97 ± 2.95 vs 22.63 ± 6.42 µm, *p* = 0.013). MT staining showed lighter blue staining of the urethral mucosa in the sham group than in the experimental group, indicating low collagen deposition (22.29 ± 3.10 vs 59.92 ± 3.33%, *p* < 0.001), as shown in Fig. 4.

**Fig. 5:**
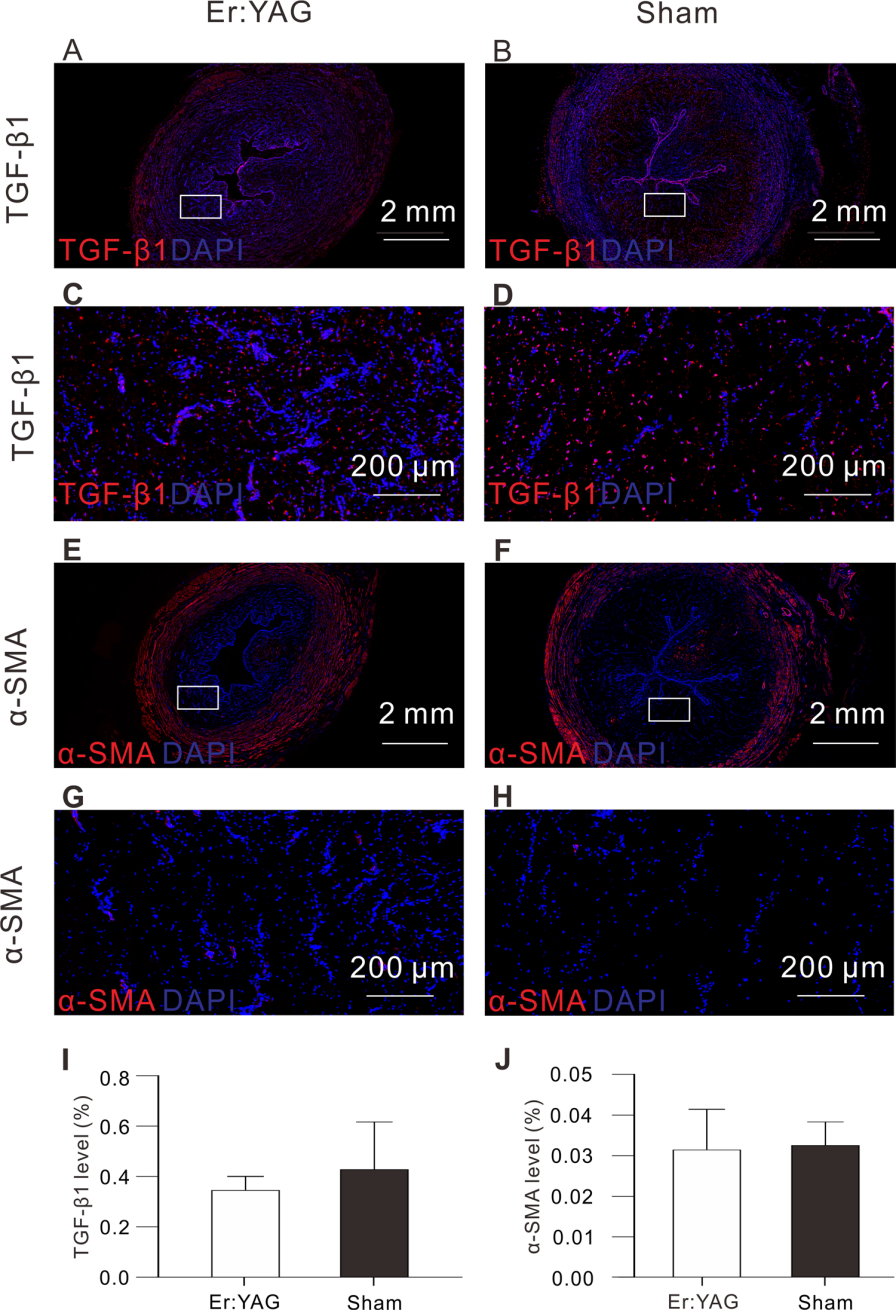
Immunohistochemistry analysis of TGF-β1 and α-SMA in the urethral mucosa at 5 weeks post-treatment. No significant differences in the expression of TGF-β1 and α-SMA were observed between the Er:YAG laser irradiation group and the sham group at 5 weeks post-treatment. Graph (**I** and **J**): Quantitative analysis of TGF-β1 and α-SMA levels in urethral tissue sections, respectively (mean ± SD)

We further investigated the effects of the Er:YAG laser on the protein expression levels of TGF-β1 and α-SMA in the urethral mucosa. As shown in Fig. 5, no significant differences in the expression of TGF-β1 and α-SMA were observed in the Er:YAG laser irradiation group compared to the sham group at 5 weeks post-treatment (0.34 ± 0.06 vs 0.43 ± 0.19%, *p* = 0.498 and 0.03 ± 0.01 vs 0.03 ± 0.01%, *p* = 0.415).

## Discussion

Er:YAG lasers are solid-state lasers that typically emit infrared light with a wavelength of 2940 nm. [21] The output of the laser is strongly absorbed by water, which is the major component of human tissue. Nonablative mode Er:YAG laser irradiation involves precisely controlled sequences of nonablative pulses that have the ability to achieve controlled heating of the deep mucosa layers to approximately 65℃ [21, 24]. At this temperature range, collagen contraction is promoted without induction of cell death, and collagen reconstruction and neocollagenesis are initiated in the treated area [21]. In this way, transvaginal nonablative mode Er:YAG laser treatment can alleviate SUI by providing reinforcement of vaginal support, which can then reduce urethral movement [19, 21, 25].SUI may be caused by an urethral hypermobility and internal sphincter deficiency (ISD). Clinically, Valsalva leak point pressure < 60 cm H_2_O is a widely accepted standard definition of ISD. There are accumulating reports indicating that intraurethral nonablative mode Er:YAG therapy has a therapeutic effect in ISD [16]. Histological data related to the effect of nonablative mode Er:YAG laser therapy in other tissues, such as the vaginal mucosa [25, 26], bone [27], skin [28], oral tissues [29, 30], and peripheral nerves [31], is currently available. However, the morphological and histological alterations in the urethra induced by this intraurethral treatment modality, which play key roles in determining its safety (especially its potential to induce urethral stricture) and efficacy, are still unknown. Although the morphological structure of the urethra is not identical in female canines and women, the urethral cavity diameters of the two species are similar, meaning that a human urethral cannula can be inserted for Er:YAG laser treatment. The urethra of the beagle also has a thick mucosa and submucosa, limiting the penetration of the Er:YAG laser to the mucosa and submucosa, which is helpful for evaluating histological changes that occur after Er: YAG laser irradiation at clinically relevant parameters. Transurethral Er:YAG laser treatment requires an urethral cannula to be placed into the urethra. In order to rule out the influence of this operation on the experimental results, a sham treatment was designed as a control group in this experiment. Urethral stricture, which is a refractory disease, can seriously affect the patient’s voiding function and quality of life. The practitioners, such as the urologists, are always concerned about this caused by the transurethral laser therapy. Our imaging results showed that the caliber of the urethral lumen in the sham group and the irradiated group was roughly the same and that narrowing was not observed in any histological samples from the irradiated group. Given the above results, it can be implied that intraurethral nonablative Er:YAG laser irradiation does not cause urethral stricture at clinically relevant parameters.

Additionally, we found that the thickness of the urethral mucosa was significantly thicker in the irradiated group than in the sham group and that the expression of collagen under the mucosa was significantly increased as well. These results are consistent with the findings of previous studies in other organs. [25, 26]. A possible mechanism for this phenomenon is that nonablative Er:YAG laser up-regulates differentiation marker genes, down-regulates immune response–related genes, and induces the expression of collagen-encoding genes. [32] More interestingly, our results showed that the urethral mucosal folds also increased in the irradiated group compared with the control group. Because many reports have shown that the urethral mucosa has the capability to improve urinary incontinence,[33] we speculate that this increase in urethral mucosal folds may, to some extent, play a role in improving the ability of the urethra to control urine flow. Theoretically, these mucosal folds can increase the resistance of the urethral lumen and mucosal seal. Due to the small number of experimental animals, further studies are needed to confirm whether this urinary control mechanism exists. Urethral fibrosis is an important pathological feature of urethral stricture. TGF-β1 signaling has been reported as the critical pathway involved in the pathology of fibrosis. To further evaluate the histological changes related to scars, we analyzed the expression of α-SMA and TGF-β1 in urethral tissue. Currently, it is generally believed that the higher the expression of TGF-β1 is, the greater the degree of scar hyperplasia [34, 35]. Additionally, α-SMA serves as a typical marker of myofibroblasts, and its expression can be upregulated by TGF-β1. The expression of α-SMA in myofibroblasts is associated with wound contraction and extracellular matrix production, thus making it important in urethral wound healing research. [35, 36] In the present study, our results revealed that the expression of TGF-β1 and α-SMA in the urethral mucosa was not significantly increased in the transurethral Er:YAG laser irradiation group compared with the sham group. Previous studies revealed that the nonablative mode of the Er:YAG laser produces collagen hyperthermia, collagen remodeling, and the synthesis of new collagen fibers. The present study found that the TGF-β1 pathway was not significantly activated. This may suggest that collagen regeneration may be mediated by other signaling mechanisms. As shown in previous studies, Er:YAG laser can induce the expression of collagen-encoding genes as described by Huth et al. [32]

### Study limitations

Although some preliminary results were obtained in our study, the present study has several limitations. First, we chose normal animals for the experiment due to the lack of widely accepted large SUI animal models. Therefore, this study does not provide evidence that nonablative erbium laser treatment can improve SUI and does not confirm the effectiveness of transurethral erbium laser treatment. Further studies are needed to explore the mechanism by which erbium laser treatment improves SUI symptoms. Second, the number of animal samples used in the experiment was relatively small. Some preliminary results obtained in this study need to be confirmed in future animal experiments with larger sample sizes. Third, due to the lack of canine urethral–specific antibodies, we used mouse and rabbit antibodies with high homology for IF, which may have had an impact on the results. Forth, in scar formation, collagen fibers may appear with the development of the pathological process. These collagen fibers can be dyed blue by MT staining. So, MT staining was used in this study. Procollagen type I could have been a better option to demonstrate fibroblast activation by the warming process. In this study, the main purpose of our research was to explore the safety of transurethral erbium laser irradiation; so this study did not evaluate the expression of procollagen type I, which was also a shortcoming of this study. In the future, we need to design more reasonable experiments to explore the role of procollagen in erbium laser treatment in the follow-up mechanism research.

## Conclusions

In summary, intraurethral nonablative Er:YAG laser irradiation at clinically relevant parameters does not cause urethral stricture in female beagle dogs. The thickening of the mucosa in the urethra and the proliferation of collagen fibers under the mucosa may be two of the mechanisms by which after erbium laser irradiation improves the symptoms of urine leakage, but further in-depth research is required.

## Abbreviations

ICS: International Continence Society
IUGA: International Urogynecology Association
SSC: Standardisation Steering Committee
UI: Urinary incontinence
SUI: Stress urinary incontinence
UUI: Urge urinary incontinence
MUI: Mixed urinary incontinence
Er:YAG: Erbium:yttrium aluminum garnet
i.v.: Intravenous injection
PFMT: Pelvic floor muscle training
TGF-β1: Transforming growth factor β1
α-SMA: α-Smooth muscle actin
IF: Immunofluorescence
H&E: Hematoxylin and eosin
MT: Masson’s trichrome
EDTA: Ethylene-diamine-tetraacetic acid
PBS: Phosphate-buffered saline
DAPI: 4ʹ,6-Diamidino-2-phenylindole
SD: Standard deviation
ISD: Intrinsic sphincter deficiency
